# Association between eating disorders and functional gastrointestinal disorders: a systematic review

**DOI:** 10.3389/fpsyg.2026.1785045

**Published:** 2026-05-07

**Authors:** Ali Saud Alsaud, Osama Homod Aldoweesh, Shaden M. Aljurayyed, Faisal Bander Alharbi, Shahad Abdalaziz Alaqeel, Haneen Sulaiman Alkhuwaildy, Farah Suliman Alsadoun, Filwah Saleh Aloufi, Hadeel Rshash Almutairi, Fahad Almatham

**Affiliations:** Department of Psychiatry, College of Medicine, Qassim University, Buraidah, Saudi Arabia

**Keywords:** anxiety, depression, eating disorders, functional gastrointestinal disorders, irritable bowel syndrome

## Abstract

**Introduction:**

Functional gastrointestinal disorders (FGIDs) and eating disorders (EDs) frequently coexist with one another. It is not unusual for a patient to present with both diseases because they share many similarities with reference to the deeper psychological factors and the connection between the gastrointestinal tract and the brain. Many studies have shown that while the occurrence of FGID in eating disorder patients is noteworthy, it varies greatly, mostly due to disparate research approaches. This review aims to synthesize 2015–2025 evidence on ED–FGID associations, prevalence patterns, bidirectionality, shared psychological mechanisms, and implications for screening and multidisciplinary management.

**Methods:**

In line with the PRISMA statement, this systematic review retrieved English-language research published within the period of 2015–2025 using a range of databases for studies related to the relationship between EDs and FGIDs. The quality of the observational studies with eligibility criteria met for data retrieval has been appraised using National Institutes of Health standards. The data has been narratively reported instead of meta-analyzed.

**Results:**

The criteria are met in nine studies published between 2016 and 2025. The studies include both the cross-sectional type and the cohort type. The overall quality of the studies leans towards being sound. The trend indicates that the co-occurrence between EDs and FGIDs is prominent, with irritable bowel syndrome being most prevalent. The two-way connection between disordered eating and GI symptoms is apparent. The impact of dietary practices, in addition to the psychological underpinning of both anxiety and depression, makes a compelling argument about the relevance of routinely screening patients with FGIDs about eating disorders.

**Conclusion:**

The reviewed article emphasizes a close link between EDs and FGIDs in terms of brain-gut overlap, wherein symptoms and eating habits mutually interact and reinforce each other. These findings underscore the importance of routine screening for eating disorders in individuals presenting with FGIDs to improve early identification and multidisciplinary management.

## 1 Introduction

Functional gastrointestinal disorders (FGIDs) are defined as a group of GI conditions that manifest as chronic and/or recurrent symptoms without any structural or biochemical abnormality. The Rome criteria standardized the diagnosis of FGIDs and emphasized the role of psychosocial factors in symptom expression (Drossman, 2016). Similarly, eating disorders (EDs) are complex conditions with multifactorial origins and strong psychosocial associations (Santomauro et al., 2021). Published by the American Psychiatric Association, the Diagnostic and Statistical Manual of Mental Disorders Fifth Edition (DSM-5) provides standardized diagnostic criteria for EDs (American Psychiatric Association, 2013).

Growing evidence found that individuals struggling with an eating disorder are three times more likely to seek attention for GI symptoms than those in a control group, suggesting an overlap between EDs and FGIDs (Winstead and Willard, 2006). According to Satherley et al. (2014), individuals suffering from gastrointestinal illnesses are more likely than healthy controls to have disordered eating behaviors, such as restrictive or irregular eating pattern. Zipfel et al. (2006) highlighted that patients with eating disorders commonly experience gastrointestinal symptoms, including nausea, bloating, and altered bowel habits, and discussed possible clinical and neurobiological mechanisms underlying these disturbances. Also, a study published in 2020 found that patients presenting with dyspepsia or gastroparesis symptoms often exhibit clinically significant Avoidant Restrictive Food Intake Disorder (ARFID)-type symptoms, which are associated with greater gastrointestinal symptom severity (Hollis et al., 2025). Furthermore, Kahi (2023) demonstrated that a substantial proportion of gastroenterology patients, particularly those with irritable bowel syndrome, screened positive for eating disorder traits when evaluated using the SCOFF questionnaire, indicating that such traits may be under-recognized in GI clinics. Collectively, these findings suggest that GI symptoms in individuals with eating disorders are not only a consequence of malnutrition but may reflect coexisting functional disorders.

Despite the growing body of evidence reported, some studies suggest that up to 60–90% of individuals with EDs experience FGID symptoms while others report lower rates (Zipfel et al., 2006). The variation of prevalence rates is a result by the differences in diagnostic criteria, populations, and assessment methods. Despite increasing recognition of this overlap, existing studies vary widely in methodology, population characteristics, and diagnostic criteria, making it difficult to draw clear clinical conclusions. Many investigations focus on single disorders (e.g., irritable bowel syndrome or anorexia nervosa) rather than examining the broader spectrum of FGIDs and EDs collectively. Furthermore, differences in the application of ROME criteria and DSM-5 classifications contribute to inconsistent prevalence estimates and uncertainty regarding the true nature of the association. As a result, clinicians lack a comprehensive synthesis of recent evidence to guide screening and management decisions. Consequently, this diversity restricts the development of clear clinical guidelines and underscores the need for systematic synthesis of current evidence. Therefore, this systematic review aims to synthesize contemporary evidence (2015–2025) regarding the association between EDs and FGIDs, evaluate prevalence patterns, examine the bidirectional nature of symptoms, explore shared psychological mechanisms, and identify implications for clinical screening and multidisciplinary management. For the purposes of this review, the term “eating disorders” includes both clinically diagnosed EDs and validated screening-based ED risk or disordered eating behaviors, as many included studies relied on screening instruments rather than formal diagnoses.

## 2 Methods

### 2.1 Literature search strategy

The systematic review was conducted in accordance with the Preferred Reporting Items for Systematic Reviews and Meta-Analyses (PRISMA) guidelines. An extensive electronic search was performed using PubMed, Web of Science, Springer, and Cochrane databases. The search was limited to studies published in English between 2015 and 2025. The time frame (2015–2025) was selected to capture studies published following the release of Rome IV criteria (2016) and DSM-5 updates, ensuring diagnostic consistency with contemporary clinical practice. The search terms used included combinations of keywords and medical subject headings (MeSH) relevant to the study objective.

### 2.2 Inclusion and exclusion criteria

Studies were included if they examined the association between eating disorders and functional gastrointestinal disorders such as irritable bowel syndrome, and gastroparesis due to its similar gastrointestinal symptoms (such as nausea, early satiety, and bloating) and its connection to restricted eating practices like ARFID, gastroparesis was added even though it is largely a motility condition rather than a conventional functional gastrointestinal disorders.; and were written in English; and utilized research designs such as cross-sectional studies, cohort studies, case–control studies, and clinical trials. Studies were excluded if they did not focus on the association between eating disorders and functional gastrointestinal disorders, or if they were case reports, systematic reviews, or meta-analyses.

### 2.3 Selection of articles and data extraction

The selection process involved multiple stages to ensure the inclusion of relevant studies. Seven independent reviewers (AA, OA, ShadA, FaiA, ShahA, HanA, FilA) initially screened the titles and abstracts of articles retrieved from the search to identify potentially relevant studies. The full text of selected articles was then reviewed independently by the same reviewers to confirm eligibility based on the predefined inclusion and exclusion criteria. Data extraction was conducted independently by the reviewers using a standardized form. Extracted data included study identification (author(s), year of publication, journal name, and title of the study); study design (study type, sample size, study duration, and study location); population (total number of participants, age range, gender distribution, inclusion and exclusion criteria); outcomes (prevalence and severity of FGIDs, quality of life measures, psychological correlations, and symptom patterns); statistical analysis (methods used, effect size, confidence intervals, and heterogeneity assessment); and conclusions (main findings, limitations, and recommendations for future research).

Disagreements during the screening and data extraction phases were resolved through discussion and consensus among all reviewers. Duplicate articles were identified and removed using Mendeley. This systematic approach ensures a comprehensive and unbiased synthesis of available evidence regarding the association between eating disorders and functional gastrointestinal disorders.

### 2.4 Quality assessment

The methodological quality of included studies was assessed using the National Institutes of Health (NIH) Quality Assessment Tool for Observational Cohort and Cross-Sectional Studies. A score between 10 and 14 denotes good quality. Scores from 7 to 9 represent fair quality, while those ranging from 0 to 6 are classified as poor quality, reflecting significant limitations.

### 2.5 Data analysis

As this study was a systematic review, no quantitative meta-analysis was performed. Data from included studies were extracted and synthesized narratively. Study characteristics, outcomes, and quality assessment results were summarized descriptively to identify patterns, consistencies, and key findings regarding the relationship between eating disorders and functional gastrointestinal disorders.

## 3 Results

### 3.1 Literature search results

**Figure 1:**
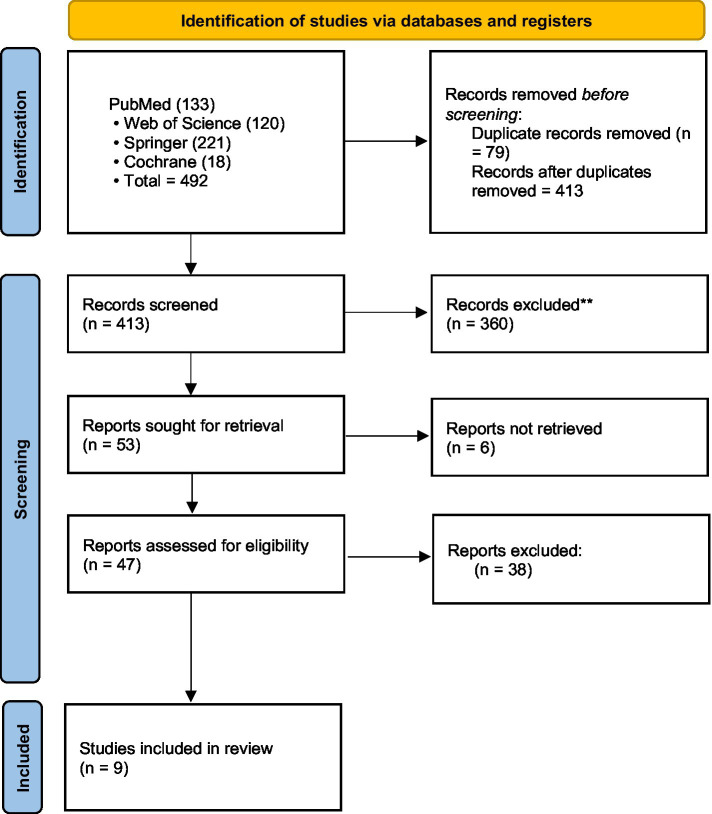
Flow chart for included studies.

The initial database search identified 492 records from PubMed (*n* = 133), Springer (*n* = 221) and WOS (*n* = 120), Cochrane (Boyd et al., 2005). After removing duplicates, 413 records remained. Screening of titles and abstracts reduced the number to 53 records. After full-text assessment, nine records met the eligibility criteria and were included in the analysis. The complete selection process is shown in Figure 1.

### 3.2 Characteristics of the included studies

Our study included nine studies (Hollis et al., 2025; Evans et al., 2023; Yang et al., 2024; Nicholas et al., 2021; Melchior et al., 2020; Spillebout et al., 2019; Reed-Knight et al., 2016; Mari et al., 2019; Newton et al., 2025), published between 2016 and 2025. Four of the included studies were cross-sectional studies and five were cohort studies. The mean age of the studies included ranged from 13.3 to 46.7 years. The sample size of the included studies ranged from 99 to 22,432 patients. Seven studies included patients with irritable bowel syndrome, and two studies included patients with gastrointestinal disorders. The sex distribution revealed no specific pattern, as the percentage of females ranged from 30.1 to 100%. The characteristics of included studies are summarized in Table 1.

**Table 1:** Characteristics of included studies.

Study ID	Year	Study design	Prevalence (number of patients)	Mean age	Disease	% Female	Aim	Conclusion
Evans et al. (2023)	2023	Cross-sectional study	225	nan	Irritable bowel syndrome	0.844	This study seeks to evaluate eating competence and disordered eating likelihood among members of online support groups for irritable bowel syndrome (IBS)	Eating competence among the sample was low at 17% while 27% was classified as likely or very likely disordered eating
Hollis et al. (2025)	2024	Cohort	107	45.4	Gastroparesis	0.841	Relationships among symptoms of gastroparesis to those of Avoidant/Restrictive Food Intake Disorder (ARFID) in patients with gastroparesis	Many (77%) patients with Gp screened positive for ARFID
Yang et al., (2024)	2024	Cohort	22,432 (participants 22,432)	27.6	Irritable bowel syndrome	1.0	To evaluate prospectively the association between maladaptive weight control/eating behaviors in females during adolescence/young adulthood	There is evidence for the potential role of early maladaptive weight control/eating behaviors in the development of adult IBS among females
Nicholas et al. (2021)	2021	Cross-sectional study	2,610 (participants 2,610)	nan	Gastrointestinal disorders	0.7	The diagnosis of avoidant restrictive food intake disorder in the presence of gastrointestinal disorders	Consideration of the interplay of a GI disorder with ARFID can add precision to case conceptualization. Food avoidance may be attempts to manage fears of aversive consequences that are augmented by a history of GI symptoms
Melchior et al. (2020)	2020	Cross-sectional study	456	42.5	Irritable bowel syndrome	0.767	To compare the prevalence of anxiety and depression states and eating disorders (EDs) between patients with irritable bowel syndrome (IBS) and healthy volunteers without IBS	The prevalence of ED assessed with positive SCOFF-F questionnaire was not significantly different between IBS patients and healthy volunteers
Spillebout et al. (2019)	2019	Cross-sectional study	731	20.1	Irritable bowel syndrome	0.301	Mental health among university students with eating disorders and irritable bowel syndrome	Students (female especially) suffer from ED and IBS, with a significant risk of co-existing ED-IBS. ED and IBS are related to multiple mental health symptoms, which could lead to negative academic consequences
Reed-Knight et al. (2016)	2016	Cohort	99 (participants 99)	18.1	Irritable bowel syndrome	0.723	Eating associated symptoms are very common in adolescents with IBS and associated with changes in eating behaviors and dietary composition	The aim of the study was to investigate if adolescents with IBS are more likely than healthy controls to experience eating associated symptoms
Mari et al. (2019)	2019	Cohort	233	13.3	Irritable bowel syndrome	0.798	The aim of this study was to assess the correlation between compliance with a low-FODMAP diet and the risk of ED behaviors among patients with IBS	In this IBS cohort, greater adherence to a low-FODMAP diet is associated with ED behavior. The implications of our study are important in clinical practice for a clinician to have a high index of suspicion of EDs in IBS patients when a high level of low-FODMAP diet achieved
Holland et al. (2025)	2025	Cohort	501 (participants 501)	46.7	Irritable bowel syndrome	0.584	The prevalence of eating disorder risk in irritable bowel syndrome	ED risk is more common in IBS than CeD or IBD. SCOFF can be used to quickly identify patients at risk of EDs particularly those with identified risk factors, such as younger patients with pre-existing mental health conditions.

### 3.3 Quality assessment

The methodological quality of included studies was assessed using the National Institutes of Health (NIH) Quality Assessment Tool for Observational Cohort and Cross-Sectional Studies. Overall, most studies were rated as good quality (scores 10–14), demonstrating clearly defined populations, validated exposure and outcome measures, and appropriate statistical analyses. However, several studies relied on self-reported measures or had small sample sizes, which may introduce bias. Detailed quality ratings are presented in (Supplementary Table S1).

### 3.4 Outcomes

#### 3.4.1 Prevalence of eating disorders in functional gastrointestinal disorders

Eating disorders are more common in individuals with gastrointestinal conditions, with studies indicating a higher prevalence of disordered eating in irritable bowel syndrome patients. Irritable bowel syndrome affects a significant portion of the population and is often associated with distressing gastrointestinal symptoms (Evans et al., 2023; Melchior et al., 2020). These symptoms frequently lead to alterations in eating behaviors, such as avoiding certain foods for no specific reason. Such restrictive eating patterns may evolve into maladaptive eating behaviors which may affect the patients’ health very badly (Evans et al., 2023; Melchior et al., 2020; Mari et al., 2019).

For example, one study found that people with IBS had more eating-related symptoms than healthy individuals. Many patients avoided certain foods for no specific reason. Some vomited after meals to reduce discomfort. These behaviors can look like Avoidant Restrictive Food Intake Disorder (ARFID). ARFID involves restrictive eating because of fear of negative reactions to food (Hollis et al., 2025).

#### 3.4.2 Bidirectional relationship: EDs and FGIDs

The relationship between eating disorders and FGIDs is likely bidirectional. Conditions like IBS and gastroparesis can make eating uncomfortable. This discomfort may lead to disordered eating behaviors. Many patients avoid foods they believe increased symptoms. Over time, this can cause nutritional deficiencies and health problems in these patients. Also, disordered eating behaviors related to restriction and fear of food intake can exacerbate gastrointestinal symptoms. Studies show that eating too little can affect how the stomach moves. This can slow digestion, as seen in gastroparesis (Evans et al., 2023; Nicholas et al., 2021).

#### 3.4.3 Impact of dietary interventions

Dietary interventions can relieve gastrointestinal symptoms and cause disordered eating behaviors. In one study, patients with IBS who adhered to a low-FODMAP diet showed a higher eating disorder symptom burden. This suggests that restrictive diets aimed at managing FGID symptoms may increase the risk of developing ED behaviors in individuals predisposed to disordered eating (Reed-Knight et al., 2016).

In gastroparesis, diet changes are a key part of treatment. However, they can increase the risk of ARFID. Many patients feel full very quickly. Nausea and vomiting are also common. These symptoms often lead people to avoid food or eat very little. First, this helps reduce discomfort. Over time, this restriction can become more severe. This can cause nutrient deficiencies and contribute to ARFID (Hollis et al., 2025; Yang et al., 2024).

#### 3.4.4 Psychological and emotional factors

Psychological factors play an important role in the relationship between eating disorders and FGIDs. Anxiety and depression are common in both groups. Emotional stress is also frequently seen. Many patients with IBS or GERD experience high levels of anxiety and depression. These feelings can affect how they eat. They can also worsen gastrointestinal symptoms. In people with FGIDs, emotional distress often leads to disordered eating behaviors. These may include binge eating or extreme food restriction (Spillebout et al., 2019).

Patients with both IBS and eating disorders often report higher stress levels. They also have a poor quality of life. This is worse than in patients with only one condition. This overlap highlights the need for special care. Treatment should address both gastrointestinal symptoms and psychological health (Melchior et al., 2020; Newton et al., 2025).

#### 3.4.5 Screening and diagnosis

Eating disorders and FGIDs often occur together. Screening for eating disorders should be routine in patients with functional gastrointestinal conditions. Tools such as the SCOFF questionnaire can help identify eating behaviors that are risky. Early detection is very important. It allows for timely and more effective treatment in these patients. This can reduce the risk of long-term problems, such as malnutrition or worsening gastrointestinal symptoms (Mari et al., 2019; Newton et al., 2025).

## 4 Discussion

The relationship between eating disorders (ED) and functional gastrointestinal disorders (FGID) is complex. These conditions often occur together in patients, which complicates diagnosis of these patients. This comprehensive review explored the relationship between these disorders by focusing on many aspects that play a role in this special relationship.

Throughout the studies included here, the prevalence of eating disorder (ED) symptoms was seen to be high in functional gastrointestinal disorders (FGIDs), particularly within Irritable Bowel Syndrome (IBS) patients. The implications of these studies suggest a two-way phenomenon wherein patients tend to avoid “trigger” foods in relation to chronic gastrointestinal symptoms or starve themselves in hopes of minimizing future episodes of sensitivity or hypersensitivity issues, while eating disorders can worsen conditions related to motility disturbances and hypersensitivity on account of a poor diet. Nutritional management can also be a part of this phenomenon wherein elimination diets related to chronic conditions such as IBS have been seen to alleviate symptoms while also potentially increasing restrictive eating behavior. Findings from studies suggest that comorbidities between mental health issues such as stress and anxiety have also been observed to be common factors that feed into exacerbations of sensitivity and perceptions related to hypersensitivity.

The results of the current literature review support existing evidence and show that there is an obvious presence of disordered eating behaviors in FGID and IBS patients. A few PubMed-indexed publications and studies show that patients diagnosed with IBS show apparently high levels of restrictive eating, food avoidance, and ED symptoms than healthy controls. In this concern, for example, authors like Satherley et al. (2014) reported high levels of disordered eating in IBS patients, strongly associated with the severity of symptoms as well as nutritional fear. In similar studies, authors like Che et al. (2025) found a high prevalence of restrictive and avoidant eating habits in patients attending tertiary care GI practices even in individuals without confirmed ED diagnoses. An inconsistency in design and assessment of different studies notwithstanding, there appears to be an overall consistency between studies regarding overlapping cases of FGID and ED symptoms, underpinning the findings and consistency between studies as non-trivial and non-methodological phenomena but genuine observations (Satherley et al., 2014; Che et al., 2025; Boyd et al., 2005).

### 4.1 Limitations unclear

This review has several limitations. First, the majority of included studies were observational, limiting causal inference. Second, many relied on self-reported screening tools rather than structured clinical interviews, increasing risk of reporting bias. Third, heterogeneity in diagnostic criteria and population characteristics may have influenced prevalence estimates.

This review has several strengths: the consideration of various study designs, the cumulative total number of subjects being quite high, and the emphasis on recent literature to make the results more applicable and generalizable. However, the majority of studies used in the review were observational in nature and could not define causality and many used self-assessed measures of ED, this may lead to issues related to reporting bias. Moreover, the longitudinal component could not provide immediacy to the relationship; this could not give much clarity to the observations made by the review regarding various analyses and tests.

This review clearly illustrates that there is a consistent convergence between eating disorders and functional gastrointestinal disorders such as IBS, based on gut-brain interactions. Disordered eating can be both precipitated by gastrointestinal symptoms and contribute to said symptoms, making it difficult to distinguish between the two. Dietary treatments, while being extremely useful, put such patients at risk. These findings clearly emphasize that screening for ED should be routine.

## Data Availability

The original contributions presented in the study are included in the article/Supplementary material, further inquiries can be directed to the corresponding author.
